# Bare Metal Stents on Resveratrol-Coated Balloons in Porcine Coronary and Peripheral Arteries

**DOI:** 10.3390/ijms222313099

**Published:** 2021-12-03

**Authors:** Stefanie Kamann, Tobias Haase, Nicola Stolzenburg, Melanie Löchel, Daniel Peters-Berg, Denise Schütt, Jörg Schnorr

**Affiliations:** 1 Department of Radiology, Experimental Radiology, Charité—University Medicine Berlin, 10117 Berlin, Germany; Tobias.haase@charite.de (T.H.); Nicola.Stolzenburg@charite.de (N.S.); Joerg.Schnorr@charite.de (J.S.); 2 InnoRa GmbH, 10115 Berlin, Germany; Melanie.Loechel@charite.de (M.L.); Daniel.Peters@innora.de (D.P.-B.); Denise.Schuett@innora.de (D.S.)

**Keywords:** drug-coated balloon catheter, stent implantation, restenosis, vascular healing, resveratrol, neovascularization

## Abstract

Balloon angioplasty and stent implantation are standard techniques to reopen stenotic vessels. Often, balloons or stents coated with cytostatic drugs are used to prevent re-occlusion of the arteries. Resveratrol, which is known for its numerous beneficial effects on cardiovascular health, is used as an antioxidant additive on paclitaxel-coated balloon catheters. What is still unclear is whether resveratrol-only balloon coating in combination with a bare metal stent (BMS) also has positive effects on vascular healing. Here, we analyzed neointimal thickening, fibrin deposition, inflammation, vasa vasorum density, and reendothelialization after implantation of BMS via a resveratrol coated balloon approach in a porcine model. In general, resveratrol treatment did not result in significantly altered responses compared to the control group in peripheral arteries. In coronary arteries, an increase in vasa vasorum density became evident three days after resveratrol treatment compared to the control group and abolished up to day 7. Significant effects of the resveratrol treatment on the fibrin score or intima-media area were transient and restricted to either peripheral or coronary arteries. In conclusion, local single-dose resveratrol treatment via a resveratrol-only coated balloon and BMS approach did not lead to adverse systemic or local effects, but also no significant beneficial effects on vascular healing were detected in the current study.

## 1. Introduction

Percutaneous transluminal angioplasty, which is often combined with stent implantation, is a standard procedure to dilate or reopen vascular stenoses. While restoring blood flow, these procedures cause mechanical stress to the vessel walls due to stretching and denudation of the endothelial layer. The vascular damage induces numerous biological processes such as inflammation, vasa vasorum neovascularization, reendothelialization, and neointima proliferation. The balance of these processes determines the extent to which vascular healing restores the physiological state of the vessel. Restenosis is one of the most common complications after frequently used angioplasty procedures, and among others is characterized by vascular smooth muscle cell (VSMC) proliferation. Cytostatic drugs such as paclitaxel or sirolimus are used as stent or balloon coatings to suppress exaggerated VSMC proliferation. As the cytostatic effects do not only act on VSMCs but also on endothelial cells (EC), delayed reendothelialization, prolonged vascular healing, and an increased risk of thrombosis may occur [1,2,3]. Thus, therapies that inhibit restenosis while supporting reendothelialization and vascular healing are a continuing therapeutic need.

Resveratrol is a naturally occurring phenol and is used as an additive on paclitaxel coated balloon catheters [4,5,6], currently introduced in selected countries and is the subject in several ongoing clinical trials (e.g., the SPORTS and SAMBA trial, evaluating the effect of resveratrol/paclitaxel coated balloon catheters peripherally in the superficial femoral artery and below the knee arteries). Resveratrol was intended to enhance the bioavailability of paclitaxel by modulating its adherence on the balloon surface and release to the vessel wall. Resveratrol is also known for its various beneficial health effects and was also the subject of several studies investigating the nutraceutical potential of resveratrol [7,8]. Systemic [9,10,11,12] and local [13] resveratrol administration was shown to reduce neointimal proliferation and improve reendothelialization after vascular injury in several animal models. However, orally administered, resveratrol has a low bioavailability. In addition, oral administration of resveratrol doses of 5 g/day has been reported to have contradictory effects and support proinflammatory processes such as tumor necrosis factor α (TNF-α) induction [14,15]. Comparison of oral versus local resveratrol administration indicated the advantages of local treatment and represents a promising method to overcome short retention time and low bioavailability [16,17]. Several systems for local delivery of resveratrol have been tested. A nanoparticle system for sustained release of locally administered resveratrol and a resveratrol–lipid conjugate were tested in vitro [18,19].

In a recent study, we demonstrated that resveratrol as a dry balloon catheter coating has beneficial effects on vascular healing in mildly, but not severely injured arteries [20].

An earlier study investigating paclitaxel-coated balloons in porcine coronary arteries showed that drug transfer from the balloon surface to the vessel wall was increased when combined with the implantation of BMS, regardless of whether the stent was pre-mounted on the coated balloon and implanted simultaneously with the drug transfer or whether the stent was implanted immediately before the treatment with the drug-coated balloon [21].

Here, we investigated the tissue transfer and effect of resveratrol as a dry balloon coating combined with BMS implantation in coronary and peripheral arteries. The current study is based on the same animal model as the previous study [20] and is intended to complement the previous findings.

## 2. Results

In a pharmacokinetic experiment, the feasibility and pharmacokinetic of resveratrol as a dry balloon catheter coating, 4–7 µg resveratrol/mm^2^ balloon surface, with a pre-mounted BMS was analyzed with focus on the amount of resveratrol transferred into the vessel wall. An efficacy and tolerance study was then performed to investigate the effects of resveratrol-coated balloon treatment in combination with BMS on vascular healing compared to the implantation of BMS alone. An overview is shown in Table 1.

### 2.1. Pharmacokinetics

In acute animals, a resveratrol content of 106 ± 58 µg/g vessel wall was determined corresponding to approximately 3% of total dose (Table 2). No resveratrol was detected in arteries 24 h after the interventional procedure, indicating a retention time of resveratrol clearly below 24 h.

### 2.2. Histological Analysis

An overview of all histological and immunohistochemical results is given in Table 3.

**Figure 1:**
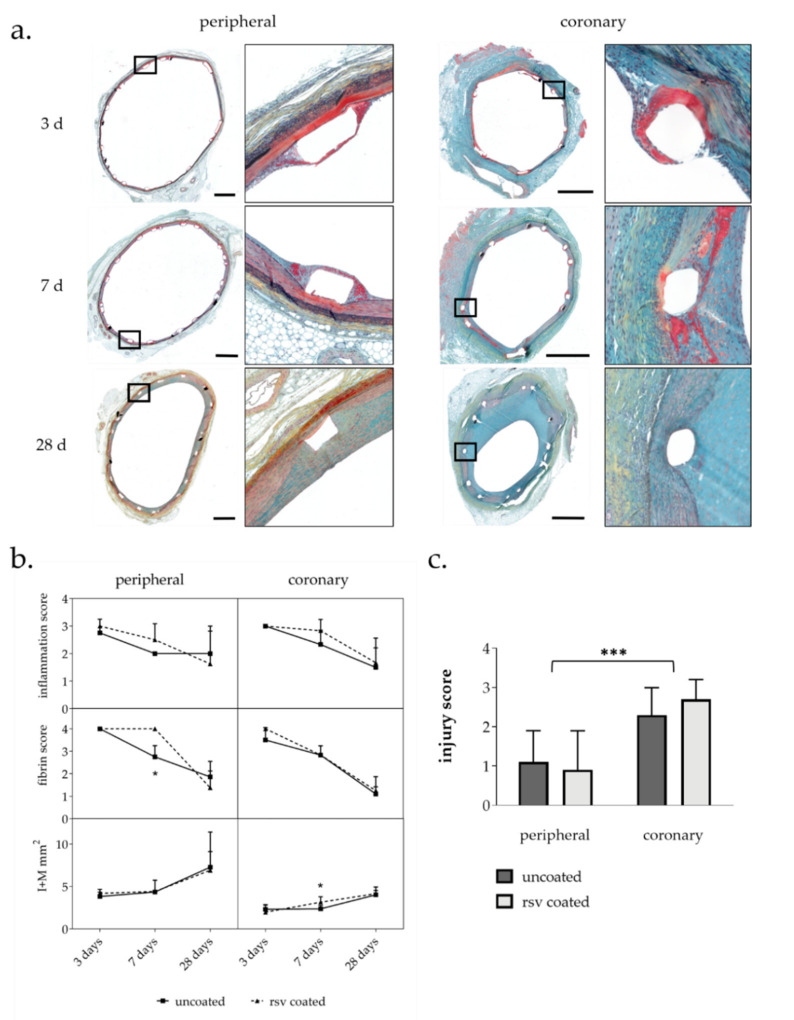
(**a**) Histological cross-sections of representative coronary and peripheral arteries 3 d, 7 d, and 28 d after BMS implantation. Movat pentachrome staining: blue—ground substance, degenerated tissue; red—smooth muscle cells; bright red—fibrin; black—elastic fiber. Bar = 1 mm. (**b**) Histological and morphometric analysis of movat pentachrome stained vessel sections. I + M intima-media area. *n* = number of arteries; *n* = 4 peripheral; *n* = 6 coronary (**c**) Injury score of peripheral and coronary arteries indicates equal treatment in both treatment groups; *n* = 42 coronary; *n* = 28 peripheral; *n*: number of vessels; values are means ± SD; *p*-value determined by Mann–Whitney test; * *p* ≤ 0.05, *** *p* ≤ 0.0001.

Movat pentachrome stain was used for histomorphometric analysis and semiquantitative detection of injury, fibrin deposition, and inflammation (Figure 1a).

#### 2.2.1. Injury Score and Morphometry

Injury scores reflect the level of mechanical damage due to overstretch in the vessel walls. In general, the injury score in our study was significantly higher in the coronaries than in the peripheral arteries (Figure 1c), indicating a higher overstretch and stent–vessel ratio in the coronaries than in the peripheral arteries. Resveratrol coating had no significant influence on the injury score (Table 2).

Vascular remodeling and neointima proliferation were analyzed by histomorphometry of treated vessels at 3-, 7-, and 28-days post intervention. Cross-section from 3 cutting levels per stented vessel segment were analyzed (Table A1). As previously described, combined intima-media area (I + M) was calculated to quantify vessel wall thickening instead of intima-media ratio. Coronary artery types (LAD, LCX, and RCA) were summarized into one coronary group for statistical analysis [20].

Overall, stent implantation led to moderate neointima formation up to 28 days. Resveratrol treatment had no influence on neointima proliferation in peripheral arteries. In coronary arteries, resveratrol led to a transient increase in intima-media area after seven days that vanished after 28 days (Figure 1b).

#### 2.2.2. Inflammation Score and Fibrin Score

The presence of inflammatory cells and fibrin allows for conclusions on the level of injury and the inflammatory status of the vessel wall and was analyzed semi-quantitatively. Fibrin as well as inflammatory cells were mainly observed in regions around and close to the stent struts. Inflammation was quantified by scoring the number of infiltrated inflammatory cells around each stent strut site.

In general, both inflammatory cell infiltration and fibrin deposition reached a maximum three days after stent implantation, which then declined. All in all, the values hardly differed between the vessel types. Vessels treated with resveratrol seemed to contain more inflammatory cells. In the resveratrol treated peripheral vessels, the fibrin level did not decrease as rapidly as in the control group and was significantly increased seven days but not 28 days after stent implantation (Figure 1b).

### 2.3. Immunohistochemical Analysis

**Figure 2:**
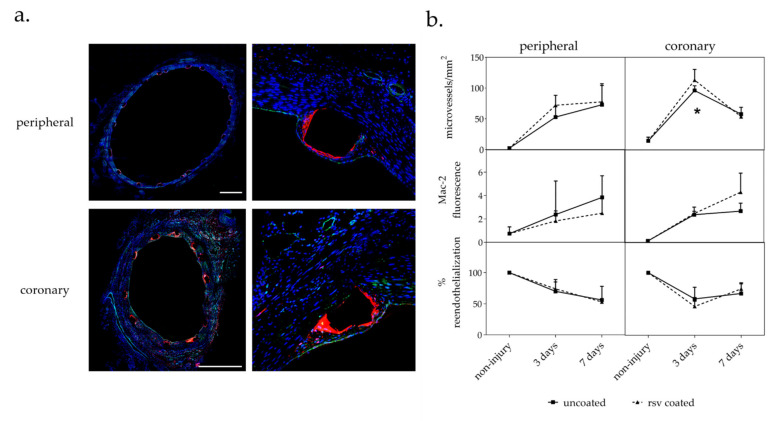
(**a**) Immunofluorescent Mac-2/CD31 staining. Double stained cross-sections of representative coronary and peripheral arteries 7 d p. i. with uncoated stent. Red: Mac-2; green: CD31, blue: dapi. Bar = 1 mm. (**b**) Immunohistological analysis. CD31/Mac-2 double staining. *n* = number of arteries; *n* = 4 peripheral; *n* = 6 coronary; values are mean ± SD; *p*-value determined by the Mann–Whitney test; * *p* ≤ 0.05.

Mac-2 positive inflammatory cells and CD31 positive endothelial cells (EC) were detected by double immunofluorescent staining (Figure 2a).

#### 2.3.1. Vasa Vasorum Neovascularization

As previously outlined, the vasa vasorum is a dynamic system of microvessels with lumen diameters of more than 0.5 mm that surrounds arteries and that modulates its density in response to the pathophysiological state of the artery. To determine neovascularization in response to stent implantation, we counted the number of microvessels on CD31 stained cross-sections per area.

In accordance to previous findings, a higher vasa vasorum baseline density was found in coronary than in peripheral arteries [20,22,23]. The number of microvessels increased in all arteries as a response to stent implantation and overstretching. Whereas the number of microvessels in peripheral arteries increased continuously within seven days after the procedure, the number of microvessels in the coronaries increased more rapidly and then decreased between days 3 and 7 after stent implantation.

Although the effect of resveratrol coating on vasa vasorum density appears to be relatively modest, it was found to significantly increase the number of microvessels at day 3 in the coronaries by 18 ± 15% (*p* = 0.041) compared to control vessels treated with uncoated devices. The same trend could be observed in the peripheral arteries (36 ± 22%), but without reaching statistical significance (Figure 2b).

#### 2.3.2. Macrophages

In addition to the inflammation score, macrophage infiltration was used as a general inflammation marker by immunohistochemically quantifying the cell surface molecule Mac-2 [24]. Mac-2 relative fluorescence was measured as mean value per section.

In general, macrophage infiltration increased up to day 7 as a response to vascular injury during stent implantation and overstretching. Resveratrol had no significant impact on the level of macrophage infiltration, although it tended to decrease the level in the peripheral arteries and, in contrast, tended to increase the level of macrophages in the more severely injured coronaries (Figure 2b).

#### 2.3.3. Reendothelialization

The level of reendothelialization provides information on the recovery of the endothelial cell monolayer following denudation during stent implantation. Immunofluorescent staining of CD31 positive cells was used to quantify endothelial cells outlining the lumen. The percentage of lumen circumference covered by CD31 cells was estimated.

In general, it was observed that the intervention denuded the lumen surface and led to a reduced number of endothelial cells compared to uninjured vessels. No resveratrol dependent effect on reendothelialization could be observed within the observational period (Figure 2b).

## 3. Discussion

In this study, we analyzed the tissue transfer rate and effects of a local single-dose intravasal resveratrol treatment via a coated balloon and stent approach in porcine coronary and peripheral arteries.

We found 3% of the initial dose transferred to the vessel wall 10 min post intervention in coronary arteries This was less but not far less than in the case of paclitaxel- or sirolimus-coating, which reduced neointimal proliferation in appropriate experiments in the same animal model. Unlike efficacious paclitaxel- and sirolimus-coatings, no resveratrol was detected in arteries 24 h after the interventional procedure, indicating a short retention time of resveratrol. A rapid metabolic conversion of resveratrol into glucuronic and sulfate derivatives [25,26] or direct interaction with its numerous identified targets [16,27] is known. It is conceivable that a part of the resveratrol was even metabolized after 10 min so that the actual initial dose transferred to the artery was possibly higher. Thus, we assumed the transfer of an actual initial amount of at least 200 µg/cm^2^ vessel surface in our efficacy study.

In general, coronary arteries showed a stronger vascular injury and neointimal proliferation than peripheral arteries. Although an overstretch of approximately 20% was targeted for all treated vessels, it is possible that the coronaries experienced more severe overstretch and/or are more sensitive to dilatation, resulting in stronger vascular injury. Nevertheless, all treated segments showed neointima formation similar to the vessels treated with uncoated balloons. Resveratrol coating did not result in significantly altered responses compared to the vessel segments not treated with resveratrol in peripheral arteries. In coronary arteries, a transient increase in neovascularization three days p. i. became evident and abolished up to day 7. Significant effects of the resveratrol treatment on the fibrin score or intima-media area were transient and restricted to either peripheral or coronary arteries.

In a previous study, we showed the feasibility of a single-dose treatment with a resveratrol coated balloon in porcine coronary and peripheral arteries. Our results indicated a modest diminishment of vascular inflammation in peripheral balloon treated arteries. Effects on neovascularization and reendothelialization were transient at three days post-intervention [20]. Since earlier studies with paclitaxel-coated balloons without resveratrol in combination with BMS demonstrated an increased drug transfer compared to drug coated balloons alone [21], we presumed that the resveratrol coated balloon plus BMS implantation might lead to prolonged resveratrol retention time at the vessel wall and enhanced anti-inflammatory effects compared to the treatment with a coated balloon alone. This, however, could not be confirmed when local resveratrol delivery was combined with stent implantation.

In other studies, resveratrol was shown to have multiple beneficial effects on vascular physiology. Most important appears to be the influence of resveratrol on nitric oxide (NO). NO production by endothelial nitric oxide synthase (eNOS) plays a central role in cardiovascular disease protection including the prevention of vascular smooth muscle cell proliferation [28]. Uncoupling of eNOS leads to the production of superoxide and peroxynitrite, leading to endothelial dysfunction [29]. In vitro and in vivo studies with orally administered resveratrol have shown that resveratrol increases eNOS expression and activity, enhances NO production, prevents eNOS uncoupling, and reduces endothelial oxidative stress [16,30,31]. In another study with a focus on the development of a drug coated stent, resveratrol was covalently attached to a stainless-steel surface [32]. It could be shown that endothelial cells grown on surface-bound resveratrol released higher amounts of nitric oxide compared to cells grown on unmodified stainless steel [32].

Anti-inflammatory and cardioprotective effects of resveratrol due to tumor necrosis factor α (TNF-α) suppression have been shown in human trials with oral doses up to 5 g per day [8,33]. At doses of more than 5 g, resveratrol turns to support proinflammatory effects due to TNF-α and NF-κb induction [15]. Interestingly, we observed an increase in vasa vasorum density after three days in coronary vessels, which is in contrast to the data from our previous balloon study. This could be due to the dose-dependent controversial effects of resveratrol.

Possible reasons for the absence of appreciable vascular protective effects of resveratrol in our stent model could be based on both under- or overdose. Potential deleterious effects due to resveratrol overdoses after local application are conceivable, but were not evident on the basis of our observations. On the other hand, resveratrol did not induce long-term beneficial effects on (e.g., reendothelialization or inflammation in the current setting). Studies that report an neointima inhibiting effect of local resveratrol treatment used experimental settings for sustained release [13] and/or resveratrol in combination with other active ingredients or excipients [13,25] and aggravates the comparison with our results. Kleinedler et al. used a combination of 150 µg resveratrol and 75 µg quercitin per cm^2^ on a drug eluting stent in rats to successfully reduce neointima formation and enhance reendothelialization [13]. Drug release in this study was biphasic and sustained [34]. Tolva et al. used the genie double balloon system to administer a resveratrol solution with a total amount of 2.8 mg resveratrol per vessel segment [25]. Unclear is how much of this effect is due to resveratrol since the carrier solution alone also significantly reduced neointima proliferation.

To conclude, local treatment with a resveratrol coated balloon with pre-mounted BMS was feasible and did not led to recognizable adverse systemic or local effects. Significant beneficial effects on vascular healing could not be detected in our animal model with our drug-coated approach. In future studies, it should be clarified whether resveratrol as an additive on drug coated balloon catheters and stents has advantages compared to other excipients in terms of vascular healing.

## 4. Materials and Methods

### 4.1. Catheter Preparation

Balloon catheters were coated with a coating solution containing 25 mg/mL resveratrol in THF/acetone/aqua (50/25/25 (m/m)). The coating procedure was undertaken with a Hamilton syringe on semi-expanded balloons with automatic rotation. Bare metal stents were pre-mounted on the back folded coated balloons with a crimper. The balloon stent approaches were sterilized by DMB Apparatebau, Wörrstadt, Germany; 38 °C/6% EO/240 min.

For the resveratrol pharmacokinetics test in porcine coronaries, B. Braun SeQuent Neo 3.5–20 mm balloon catheters were coated with 4.0 ± 0.3 µg/mm^2^ resveratrol. Fortimedix, Coro Large (KaOn CoCr), 14.3 mm stents were then pre-mounted on the back folded coated balloons.

For the resveratrol efficacy study, the balloons were coated with 6.8 ± 0.2 µg/mm^2^ resveratrol. Fortimedix Kaon coro large 14 mm length stents on Falcon Bravo 3.5–20 mm, Rx 0.014“, 145 cm shaft balloon catheters for the application in coronary arteries and stainless steel B. Braun 6–7 mm diameter, 17.8 mm length stents on ClearStream PSC2 6.0–60 mm or 7.0–60 mm, and OTW 0.035“ balloon catheters for the application in peripheral arteries were used.

### 4.2. Animal Experiments

To analyze the pharmacokinetics of resveratrol, four domestic pigs, three months old with body weights of 24.0–30 kg, underwent balloon angioplasty and stent implantation in three coronary arteries (RCA, LAD, LCX) with resveratrol-coated balloon catheters.

To analyze the efficacy of resveratrol, 16 domestic pigs, three months old, with body weights of 24.0–30.5 kg, underwent balloon angioplasty and stent implantation in three coronary and two peripheral arteries, each with uncoated and resveratrol coated balloon catheters.

Details have been described previously in [35]. Briefly, two days before treatment, dual-antiplatelet therapy (75 mg Clopidogrel and 100 mg acetylsalicylic acid) was administered. Long-acting Verapamil was given within 24 h prior to the procedure to reduce vascular spasm during the procedure. The pigs were sedated before general anesthesia was induced. Blood pressure was recorded before and after the treatment. Throughout the procedure, the electrocardiogram, arterial oxygen saturation (SpO2), and temperature were monitored. Access was provided through an external carotid artery. Heparin 5000 IU and 250 mg lysine acetylsalicylate were administered intra-arterially. Vessel segments in coronary or iliac/femoral arteries were selected. The balloons were deployed as indicated and inflated for 60 s with 8 to 14 atm to achieve a balloon to artery diameter ratio of approximately 1.2.

Pigs were euthanized at 10 min or 24 h (pharmacokinetic test) and three days, seven days, or 28 days (efficacy study) after balloon treatment. For euthanasia, 10 mL super-saturated potassium chloride (25%) was injected intravenously in deep anesthesia.

### 4.3. Resveratrol Analysis by HPLC-UV

Used balloons were collected into vials for resveratrol analysis. Ethanol was added. The vials were firmly closed and intensely shaken in a test tube shaker for at least 30 s.

Treated coronary artery segments were dissected, placed in pre-weighted vials, and kept frozen until drug content analysis. For extraction, ethanol was added to the dissected segments to achieve an ethanol concentration of >50%. Samples were homogenized (Precelly 24 Dual Homogenizer, PEQLAB Biotechnologie GmbH, Erlangen, Germany).

All samples were extracted by ultrasonication at room temperature for 30 min followed by centrifugation at 17,500× *g* for 10 min.

Resveratrol extracted from balloon catheters and tissue samples was determined by HPLC with UV detection. Column: C18, 5 μm, 25 cm × 4.6 mm. Mobile phase: 45% phosphate buffer 0.005 M (pH 3.5) and 55% acetonitrile, 1 mL/min. Detection: 230 nm.

Retention time: 3.97 ± 0.02 min. Detection limit: <1 µg/mL. A standard solution was injected during the same run (resveratrol concentration 10 µg/mL).

### 4.4. Histochemistry and Morphometry

Treated vessel segments were dissected, fixed in 4% formalin solution for 24 h, and embedded in polymethyl-methacrylate (PMMA). To cut each stent segment in three levels, the PMMA blocks were cut in three segments with a coping saw and re-embedded in PMMA. Tissue sections of 4 µm thickness were generated on a rotary microtome with a tungsten carbide knife. Three sections of each level were stained by Movat pentachrome staining for histomorphometric analysis (Table A1). Microscopic images were acquired with AxioObserver.Z1 (Carl Zeiss Vision GmbH, Jena, Germany) and analyzed using ImageJ software (National Institutes of Health, Bethesda, USAA). Due to frequent IEL rupture in the coronaries, neointima formation was quantified by intima-media area (I + M) instead of intima/media ratio (I/M).

We analyzed injury score, inflammation score, and fibrin score as previously described. All scores were assessed semi quantitatively for each stent strut site. Injury score: (0) IEL intact; endothelium typically denuded; media compressed but not lacerated; (1) IEL lacerated; media typically compressed but not lacerated; (2) IEL lacerated; media visibly lacerated; EEL intact but compressed; and (3) EEL lacerated; typical large lacerations of media extending through the external elastic lamina; coil wires sometimes residing in adventitia. Inflammation score: (0) no inflammatory cells surrounding the strut; (1) light, noncircumferential lymphohistocytic infiltrate surrounding the strut; (2) localized, moderate to dense cellular aggregate surrounding the strut noncircumferentially; and (3) circumferential dense lymphohistiocytic cell infiltration of the strut. Fibrin score: (0) No fibrin present around strut; (1) fibrin deposition in <25% around the strut; (2) fibrin deposition 25–50% around strut; (3) fibrin deposition 50–75% around strut; and (4) fibrin deposition 100% around strut. All scores were assessed semiquantitatively for each stent strut site. The worst value per cross-section was used for further calculation.

### 4.5. Immunohistochemistry

Double immunofluorescent staining of Mac-2 (clone M3/38, Cedarlane, Burlington, Ontario, Canada) and CD31/PECAM-1 (clone M-20-R, Santa Cruz Biotechnology, Dallas, Texas, US) was conducted to determine neovascularization, infiltration of macrophages into the vessel wall, and reendothelialization of the vessel lumen.

Sections were deplastinated in 2-methoxyethyl acetate. Antigen retrieval was carried out by boiling in citrate buffer pH 6 for 30 min. Sections were incubated with primary antibodies (1:100) in Dako REAL antibody diluent (Agilent, Santa Clara, California) at 4 °C overnight followed by incubation of AlexaFluor-labeled secondary antibodies (Invitrogen, Carlsbad, California, USA), 1:200, 45 min at room temperature.

Microscopic images were acquired with AxioObserver.Z1 and Apotome (Carl Zeiss Vision GmbH, Jena, Germany) and analyzed using ImageJ software (National Institutes of Health, Bethesda, USA). The relative fluorescence of Mac-2 labeled cells was quantified. Reendothelialization was determined by labeling CD31 positive cells outlining the lumen and measurement of lumen circumference covered by CD31 cells in increments of 5 percent. Neovascularization was analyzed by counting CD31 labeled microvessels in the adventitia per area.

### 4.6. Statistical Analysis

Quantitative and semiquantitative parameters were compared by the Kruskall–Wallis test for nonparametric values followed by the Mann–Whitney test to determine statistical significance between treatment groups at each time point using Prism 8 (GraphPad Software, San Diego, California). *p* levels of <0.05 were considered as statistically significant. Data are presented as mean value ± SD.

## Figures and Tables

**Table 1:** Overview of studies performed.

Study	Number of Animals	Survival Time	Vessel Type	Number of Vessels ^1^	Total Dose on Balloon ^2^	Dose per mm^2^ Balloon Surface
pharmacokinetic	2	10 min	coronary	6	1010 ± 74 µg	4.0 ± 0.3 µg/mm^2^
	2	1 d	coronary	6	1010 ± 74 µg	4.0 ± 0.3 µg/mm^2^
efficacy	4	3 d	coronary	6	1636 ± 29 µg	6.5 ± 0.1 µg/mm^2^
				6	-	-
			peripheral	4	9748 ± 396 µg	6.9 ± 0.3 µg/mm^2^
				4	-	-
	4	7 d	coronary	6	1636 ± 29 µg	6.5 ± 0.1 µg/mm^2^
				6	-	-
			peripheral	4	9748 ± 396 µg	6.9 ± 0.3 µg/mm^2^
				4	-	-
	8	28 d	coronary	12	1636 ± 29 µg	6.5 ± 0.1 µg/mm^2^
				12	-	-
			peripheral	8	9748 ± 396 µg	6.9 ± 0.3 µg/mm^2^
				8	-	-

^1^ A maximum of two peripheral and three coronary arteries can be treated per animal, ^2^ Doses in the peripheral groups are mean values from two balloon sizes.

**Table 2:** Resveratrol content in coronary arteries after treatment with resveratrol-coated balloon catheters with pre-mounted stents. SD: standard deviation.

Survival	Vessel	Balloon Inflation Pressure ATM	µg Total	µg/g Tissue	% of Total Dose
10 min	RCA	10	37	150	3.7
	LAD	8	19	79	1.9
	LCX	10	48	161	4.8
	RCA	12	24	92	2.4
	LAD	10	3	9	0.3
	LCX	10	40	147	4.0
mean ± SD			29 ± 16	106 ± 58	2.8 ± 1.6

ATM = atmosphere, standard unit of pressure; SD = standard deviation.

**Table 3:** Summary of results. *n*-value: number of arteries included; I + M: Intima-media area; Macrophages: relative fluorescence of immunofluorescent stained Mac-2; values are mean ± SD; *p*-value determined by the Mann–Whitney test; * *p* ≤ 0.05.

Survival	Analysis Parameter	Peripheral		*p*-Value	Coronary ^1^		*p*-Value
		Uncoated	RSV Coated		Uncoated	RSV Coated	
3 d	*n*-value	4	4		6	6	
	Injury score	0.08 ± 0.03	0.06 ± 0.08	0.428	0.92 ± 0.73	0.91 ± 0.29	0.999
	Inflammation score	2.05 ± 0.30	2.20 ± 0.17	0.999	2.45 ± 0.25	1.97 ± 0.39	0.999
	Fibrin score	3.50 ± 0.26	3.28 ± 0.22	0.999	2.88 ± 0.50	3.29 ± 0.26	0.182
	I + M (mm^2^)	3.82 ± 0.51	4.19 ± 0.47	0.486	2.31 ± 0.50	1.97 ± 0.90	0.310
	Macrophages	2.37 ± 2.87	1.83 ± 0.86	0.686	2.38 ± 0.29	2.48 ± 0.55	0.818
	% reendothelialization	70 ± 19	74 ± 11	0.971	58 ± 19	46 ± 8	0.192
	neovascularization	52.98 ± 16.61	72.08 ± 16.17	0.343	96.25 ± 7.66	113.19 ± 17.26	0.041 *
7 d	*n*-value	4	4		6	6	
	Injury score	0.18 ± 0.16	0.01 ± 0.03	0.485	0.98 ± 0.40	1.11 ± 0.43	0.197
	Inflammation score	1.44 ± 0.33	1.88 ± 0.37	0.429	1.61 ± 0.33	2.08 ± 0.57	0.242
	Fibrin score	1.92 ± 0.43	2.93 ± 0.50	0.029 *	2.11 ± 0.42	2.09 ± 0.45	0.999
	I + M (mm^2^)	4.35 ± 1.39	4.41 ± 1.32	0.886	2.37 ± 0.40	3.15 ± 0.65	0.015 *
	Macrophages	3.84 ± 1.85	2.49 ± 1.47	0.486	2.68 ± 0.69	4.30 ± 1.62	0.114
	% reendothelialization	56 ± 22	53 ± 25	0.914	67 ± 17	74 ± 8	0.914
	neovascularization	72.97 ± 34.07	77.67 ± 26.49	0.886	58.04 ± 11.27	53.89 ± 3.86	0.761
28 d	*n*-value	7	8		10	12	
	Injury score	0.29 ± 0.42	0.25 ± 0.28	0.814	1.53 ± 0.41	1.79 ± 0.51	0.213
	Inflammation score	1.21 ± 0.58	0.65 ± 0.50	0.587	0.90 ± 0.45	1.02 ± 0.70	0.839
	Fibrin score	0.70 ± 0.35	0.64 ± 0.30	0.215	0.64 ± 0.20	0.54 ± 0.16	0.857
	I + M (mm^2^)	7.27 ± 1.85	6.91 ± 4.50	0.232	4.01 ± 0.52	4.15 ± 0.79	0.892

^1^ Data pool of left anterior descending artery (LAD), left circumflex coronary artery (LCX), and right coronary artery (RCA).

## Data Availability

The data presented in this study are available on request from the corresponding author. The data are not publicly available.

## Appendix A

**Table A1:** Number of animals, arteries, and sections included in analysis. Movat stained sections were used to analyze injury score, inflammation score, fibrin score, and I + M. Mac-2/CD31 double stained sections were used to analyze macrophages, reendothelialization, and neovascularization.

Animals	Survival	Artery Type	Staining Method	Vessels (Uncoated, RSV Coated)	Section Levels	Sections per Level	Sections Total
**4**	3 d	peripheral	Movat	4, 4	1	1	8
			Mac-2/CD31	4, 4	1	2	16
		coronary	Movat	6, 6	1	1	12
			Mac-2/CD31	6, 6	up to 3	up to 4	43
**4**	7 d	peripheral	Movat	4, 4	1	1	8
			Mac-2/CD31	4, 4	1	2	16
		coronary	Movat	6, 6	1	1	12
			Mac-2/CD31	4, 6	up to 3	up to 4	32
**8**	28 d	peripheral	Movat	7, 8	1	1	15
		coronary	Movat	11, 12	1	1	23
